# Differential expression of microRNAs in mouse pain models

**DOI:** 10.1186/1744-8069-7-17

**Published:** 2011-03-07

**Authors:** Ricardo Kusuda, Flaviane Cadetti, Maria I Ravanelli, Thais A Sousa, Sônia Zanon, Fernando L De Lucca, Guilherme Lucas

**Affiliations:** 1 Department of Physiology, Ribeirão Preto School of Medicine, University of São Paulo, Ribeirão Preto, Brazil; 2 Department of Neurology, Ribeirão Preto School of Medicine, University of São Paulo, Ribeirão Preto, Brazil; 3 Department of Biochemistry and Immunology, Ribeirão Preto School of Medicine, University of São Paulo, Ribeirão Preto, Brazil

## Abstract

**Background:**

MicroRNAs (miRNAs) are short non-coding RNAs that inhibit translation of target genes by binding to their mRNAs. The expression of numerous brain-specific miRNAs with a high degree of temporal and spatial specificity suggests that miRNAs play an important role in gene regulation in health and disease. Here we investigate the time course gene expression profile of miR-1, -16, and -206 in mouse dorsal root ganglion (DRG), and spinal cord dorsal horn under inflammatory and neuropathic pain conditions as well as following acute noxious stimulation.

**Results:**

Quantitative real-time polymerase chain reaction analyses showed that the mature form of miR-1, -16 and -206, is expressed in DRG and the dorsal horn of the spinal cord. Moreover, CFA-induced inflammation significantly reduced miRs-1 and -16 expression in DRG whereas miR-206 was downregulated in a time dependent manner. Conversely, in the spinal dorsal horn all three miRNAs monitored were upregulated. After sciatic nerve partial ligation, miR-1 and -206 were downregulated in DRG with no change in the spinal dorsal horn. On the other hand, axotomy increases the relative expression of miR-1, -16, and 206 in a time-dependent fashion while in the dorsal horn there was a significant downregulation of miR-1. Acute noxious stimulation with capsaicin also increased the expression of miR-1 and -16 in DRG cells but, on the other hand, in the spinal dorsal horn only a high dose of capsaicin was able to downregulate miR-206 expression.

**Conclusions:**

Our results indicate that miRNAs may participate in the regulatory mechanisms of genes associated with the pathophysiology of chronic pain as well as the nociceptive processing following acute noxious stimulation. We found substantial evidence that miRNAs are differentially regulated in DRG and the dorsal horn of the spinal cord under different pain states. Therefore, miRNA expression in the nociceptive system shows not only temporal and spatial specificity but is also stimulus-dependent.

## Background

MicroRNAs (miRNAs) are endogenously expressed short non-coding RNAs thought to inhibit protein translation through binding to a target complementary mRNA [1-6]. Thus, the encoded genetic information is not only transcribed and translated into proteins but also regulates these processes through miRNA sequence-guided interactions with the related miRNA [2,7-9]. Expression analysis of miRNAs has been widely used to monitor tissue-specific miRNA expression and regulatory changes in developmental stages, cell types and tissues [1,10,11]. Tissue and temporal specificity suggest that miRNAs sequences have an organ and/or cell type-specific function [12-16]. Furthermore, abnormal patterns of miRNA expression have also been found in many disease states where both increased and decreased expression of miRNAs have been described [16,17]. The first experimental reports addressing the involvement of miRNAs in the nociceptive system clearly indicate that inflammatory muscle pain [18], and peripheral nerve injury [19] modify the expression profile of a number of miRNAs in trigeminal and dorsal root ganglion, respectively. A recent work provided evidence that miRNAs regulate the expression of several transcripts associated with inflammatory pain [20]. Indeed, the nociceptive system is substantially modified in response to tissue damage, inflammation or injury to the nervous system where changes in gene expression patterns are a marked molecular mechanism underlying the development and maintenance of chronic pain [21-25]. Hence, transcriptional changes can dramatically alter the phenotypic profile and function of neurons and glia cells in the dorsal root ganglion and spinal cord dorsal horn, where nociceptive messages are primarily released to the central nervous system [21,22]. However, our understanding on the mechanisms regulating post-transcriptional machinery remains very limited. In the present study we tested the hypothesis that in addition to temporal or spatial-specificity miRNA expression is also stimulus-dependent in the nociceptive system. In the present study the criteria to select four miRNAs were their reported expression in the mouse nervous system [10,26] and/or their predicted pain-related target genes, such as brain-derived neurotrophic factor, mitogen activated protein kinase, phospholipase A2, and opioid receptor from in silico investigation [27,28]. Therefore, we investigated the temporal, spatial and stimulus-dependent specificity of miRNAs by monitoring the time-course expression of miR-1, miR-16, miR-122a, and miR-206 in mouse DRG and spinal cord dorsal horn under inflammatory and neuropathic pain states as well as after acute nociceptive stimulation.

## Methods

### Animals

Adult Balb/c mice (20-25 g) were housed 4-5 per cage on a 12 hours light/dark cycles (lights on at 6 A.M.) and kept at 25°C ± 1°C. Food and water were available *ad libitum*. Behavioral experiments were performed between 9 A.M. and 4 P.M. The experimental procedures performed on animals were approved by the Ethical Committee for Animal Experimentation of Ribeirão Preto School of Medicine, University of São Paulo and followed the International Association for the Study of Pain guidelines for investigations of experimental pain in conscious animals [29].

### Chronic inflammatory pain model

Tissue inflammation was produced by injecting 20 μL of CFA-complete Freund's adjuvant (Sigma, St. Louis, MO) subcutaneously in the dorsal aspect of the left hind-paw whereas mineral oil (Sigma) was used as control. Paw withdrawal thresholds to mechanical stimuli were assessed 12 h, 1, 3 and 7 days post-injection. At the completion of behavioral testing, mice were euthanized. Control animals were euthanized 12 h post-injection.

### Neuropathic pain model

Nerve injury was performed in anesthetized mice (ketamine and xylazine, 60 and 8 mg/kg, respectively) by tying a tight ligature with 8-0 silk wire around approximately one-third to one-half of the diameter of the left sciatic nerve [30]. Sham-operated animals had the left sciatic nerve exposed, but not ligated. After surgery nerve-injured animals were randomly separated in 4 groups and the development of tactile stimulus-induced neuropathic pain hypersensitivity was assessed at 1, 3, 7 and 14 days post-injury. By the end of the behavioral assay, mice were euthanized. Sham-operated control animals were euthanized 12 h post-injection.

### Axotomy

Animals were anesthetized with ketamine (60 mg/kg) and xylazine (8 mg/kg), and had the left sciatic nerve transected. A segment of approximately 1 mm was removed and the stumps were tightly ligated with 8-0 silk wire. Sham-operated animals had the sciatic nerve exposed but not sectioned. Nerve-injuried animals were separated in 3 groups and euthanized 1, 3, and 7 days post-lesion. Sham-operated animals were killed 24 hours after surgery. The left L4-L5 DRG and lumbar spinal dorsal horn were harvested immediately after euthanasia and processed for total RNA extraction.

### Acute noxious stimulation

Acute pain was induced by subcutaneous injection of capsaicin in the dorsal aspect of the left hindpaw. Two doses of capsaicin were tested, 2 and 10 μg/20 μL. Control animals were injected with vehicle (89.5% saline, 10% ethanol, 0.5% Tween-80). Animals were euthanized 10 minutes post injection and DRG and the lumbar spinal cord dorsal horn dissected out for RNA extraction.

### Behavior analysis

Mechanical hypersensitivity was assessed before and after the injection of CFA or nerve injury by measuring the paw withdrawal threshold in response to probing calibrated Semmes-Weinstein monofilaments (von Frey hairs; Stoelting, Wood Dale, IL). Animals were placed on an elevated meshed grid which allowed full access to the ventral aspect of the hindpaws. A logarithmic series of 9 filaments were applied to the left hindpaw to determine the threshold stiffness required for 50% paw withdrawal according to the non-parametric method of Dixon [31] as described by Chaplan et al. [32]. This behavioral analysis ensured that all animals selected to the miRNA expression assay developed mechanical hypersensitivity over the entire period of investigation in the inflammatory and neuropathic pain models. In the acute pain model, nocifensive behavior was monitored as time spent biting/licking capsaicin or vehicle-injected paw for 10 min.

### Tissue dissection

Animals were euthanized by cervical dislocation and the left DRG (L4-L5) as well as the lumbar (L4-L6) spinal cord were dissected out. Next, the spinal cord was further dissected in PBS (4°C) by removing only the left superior quadrant of the spinal cord. Then, the tissues were rapidly homogenized in Trizol reagent at 4°C and frozen at -80°C for further processing.

### Multiplexing reverse transcriptase reaction

Total RNA from DRG and the dorsal horn of the spinal cord was isolated using Trizol^® ^reagent (Invitrogen) according to the manufacture's instruction. RNA quality and quantity were assessed using a spectrophotometer (Eppendorf BioPhotometer plus). For multiplexing reverse transcriptase reactions we used TaqMan microRNA Reverse Transcription kit with specific primers for miR-1, -16, -122a, -206, and snoR-202 following protocol provided by the manufacture (Applied Biosystems).

### Real-time RT-PCR

To quantify miRNAs by real-time RT- PCR we used TaqMan^® ^Universal PCR Master Mix, No AmpErase^® ^UNG (Applied Biosystems). Amplification was performed according to the manufacture's standard protocol. PCR primers and probes for amplification of the mouse mature miRNAs were specifically design for miR-1, -16, -122a, -206, and snoR-202 (Applied Biosystems). RT-PCR analysis was performed on an ABI5500HT instrument (ABI Inc.). All reactions were run in duplicate. The relative quantity of each miRNA in the tissues was calculated using the equation RQ = 2^-ΔΔ*CT *^[33]. SnoR-202 was measured by the same method and remained stable along the tested time period (data not shown). Therefore, snoR-202 was used for normalization as the internal control gene whereas the calibrator was the mean threshold cycle (*C*_T_) value for each control group associated with their respective pain model.

## Results

**Figure 1:**
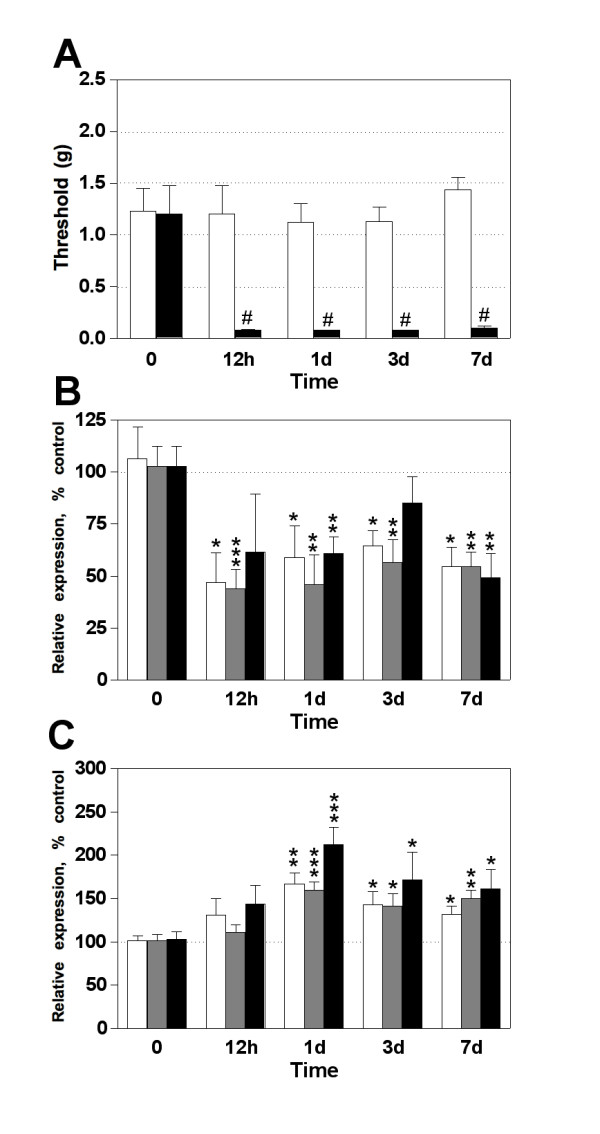
**Expression profile of mature microRNAs in DRG and the dorsal horn of the spinal cord during peripheral inflammation**. (**A**) CFA injection induced mechanical hypersensitivity 12 hours, 1, 3 and 7 days post-injection (black bars) compared to the basal values (white bars). Mineral-oil injected mice showed no threshold changes from day 1 to 7 post-injection. (**B**) In DRG, miR-1 (white bars) and -16 (gray bars) were significantly downregulated under the entire inflammatory period. However, miR-206 (black bars) was down regulated only on day 1 and 7 post-injection. (**C**) In the spinal dorsal horn there was no change in the early inflammatory process (12 h post-injection) whereas the relative expression of miRs-1, -16 and -206 showed a significant increase at day 1, 3 and 7 post-injection. Bars represent mean ± SEM. # *p *< 0.001 for CFA injected animals compared to mineral oil injected mice, n = 6 - 8 per group; Student's *t*-test. * *p *< 0.05, ** *p *< 0.01, and *** *p *< 0.001 for each miRNA of the treated group compared to mineral-oil injected animals and euthanized 12 h post-injection (time 0), n = 6-8 per group; Student's *t*-test.

We first monitored the mechanical sensitivity at different times after subcutaneous CFA administration, ensuring that all animals selected to the gene expression assays developed tactile mechanical hypersensitivity over the entire period of investigation (Figure 1A). Thereafter, we were able to detect miR-1, -16 and -206, but not miR-122a even after 40 cycles. CFA injection induced a significant downregulation of miR-1, -16 in DRG as early as 12 h persisting until 7 days post-injection (Figure 1B). However, the expression of miR-206 showed an irregular profile being downregulated at days 1 and 7, but returned to normal levels by day 3 post-injection (Figure 1B). On the contrary, in the dorsal horn of the spinal cord the expression of miR-1, -16, and -206 showed a significant increase at day 1, 3 and 7 but not in the initial inflammatory process (Figure 1C).

**Figure 2:**
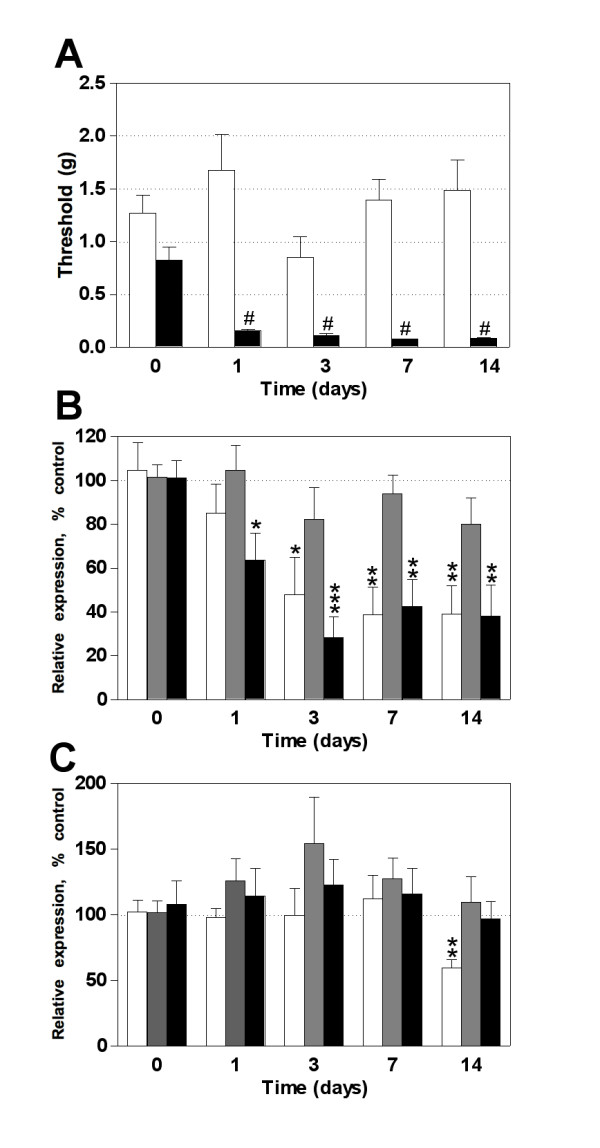
**The effect of partial sciatic nerve ligation on miRNA expression in DRG and spinal cord dorsal horn**. (**A**) Mice submitted to nerve lesion developed tactile stimulus-induced hypersensitivity at 1, 3, 7 and 14 days post-surgery (black bars) whereas sham-operated animals (white bars) showed no change on mechanical threshold. (**B**) In DRG, miR-206 (black bars) was downregulated at all time points investigated whereas miR-1 reduced relative expression (white bars) occurred only after the day 3 post-surgery. No significant change was detected for miR-16 (gray bars). (**C**) In spinal cord dorsal horn, there was no modification in the expression profile for any miRNA studied. Bars represent mean ± SEM. # *p *< 0.001 for nerve injured animals compared to sham-operated mice, n = 6 - 8 per group; Student's *t*-test. * *p *< 0.05, ** *p *< 0.01, and *** *p *< 0.001 for each miRNA expression in nerve injuried group compared to sham-operated control animals euthanized 24 h after surgery (time 0), n = 6 - 8 per group, Student's *t*-test.

**Figure 3:**
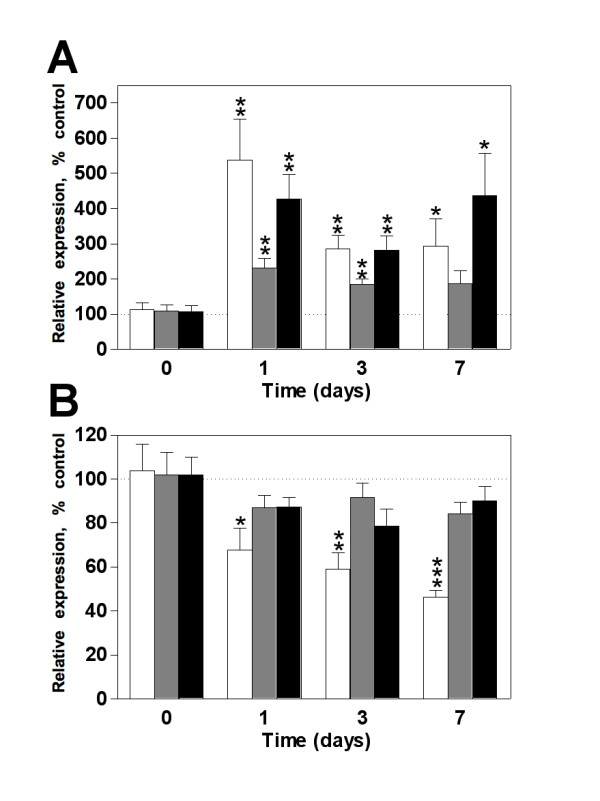
**Expression profile of microRNAs in DRG and spinal dorsal horn after sciatic nerve axotomy**. (**A**) MiR-1 (white bars) and -206 (black bars) showed a robust upregulation in the dorsal root ganglion whereas miR-16 (gray bars) relative expression also increased, but only on days 1 and 3 post-injury returning to its normal values on day 7 post-nerve lesion. (**B**) in the dorsal horn of the spinal cord, miR-1 was downregulated over the entire period of investigation and no change was observed for miR-16 and -206. Bars represent mean ± SEM. * *p *< 0.05, ** *p *< 0.01, and *** *p *< 0.001 for each miRNA expression in axotomized group compared to sham-operated control animals euthanized 24 h after surgery (time 0), n = 6 - 8 per group, Student's *t*-test.

We next analyzed the detectable levels of the three miRNAs in DRG and the spinal dorsal horn of animals submitted to partial ligation of the sciatic nerve. This model allowed us to monitor the development of tactile stimulus-induced neuropathic pain hypersensitivity. Peripheral nerve lesion induced a marked mechanical allodynia from day 1 to 14 post-injury whereas sham-operated animals showed no change in mechanical sensitivity (Figure 2A). The expression profile of miR-1 in DRG showed no change at day 1 after nerve injury but a significant downregulation at day 3, 7 and 14 post-surgery (Figure 2B). Conversely, miR-16 showed no difference in the expression level over the entire period of study. However, the expression pattern of miR-206 was similar to miR-1. A remarkable downregulation was observed as early as day 1, persisting at days 3, 7 and 14 (Figure 2B). In the spinal cord dorsal horn no change was observed in the expression profile of any miRNAs investigated (Figure 2C). It is well characterized that pain from different origins may induce specific phenotypic changes in DRG and the dorsal horn of the spinal cord. Then, we used another model of neuropathic pain by axotomizing the sciatic nerve. Opposite to the results observed in the partial nerve lesion model, complete peripheral nerve section induced an upregulation of miR-1, -16 and -206 in DRG at days 1, 3 and 7 post-injury (Figure 3A). On the other hand, miR-1 showed a significant decrease in the spinal dorsal horn from day 1 to day 7 whereas no change was observed in the expression of miR-16 and -206 (Figure 3B).

**Figure 4:**
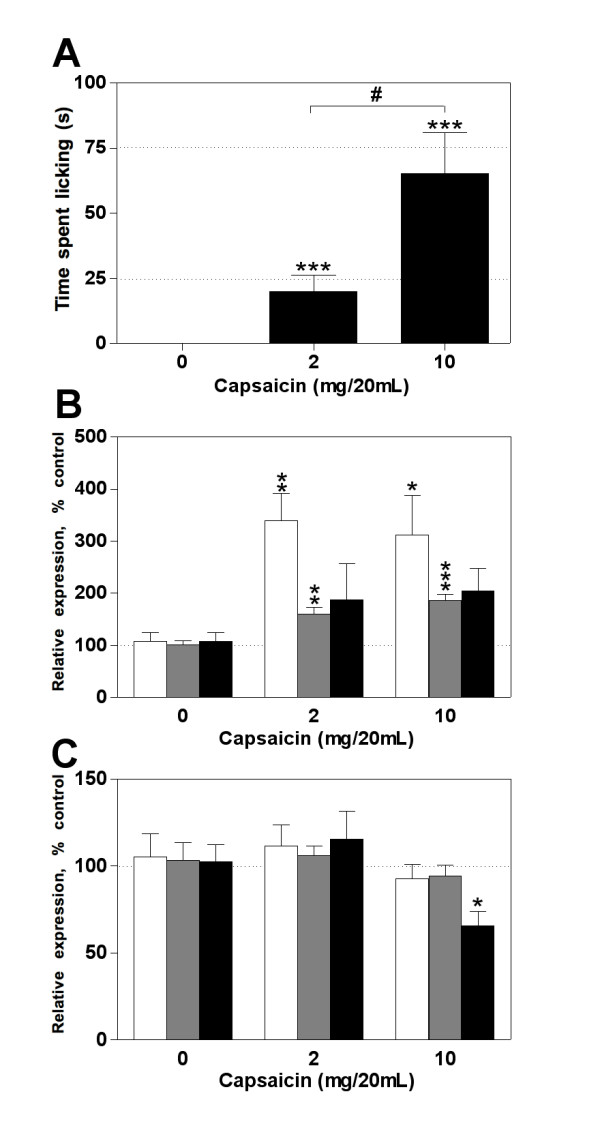
**Effect of acute noxious stimulation on miRNA expression in DRG and the dorsal horn of the spinal**. (**A**) Capsaicin induced a concentration-dependent nocifensive behavior measured by the time spent licking the injected hindpaw. Control group injected with vehicle showed no alteration on the behavior response. (**B**) Ten minutes after stimulation, miR-1 (white bars) and -16 (gray bars), but not miR-206 (black bars), showed an increased expression in DRG, however, this upregulation was not concentration-dependent as observed in the behavior test. (**C**) In the spinal dorsal horn, 2 μg of capsaicin had no effect on miRNAs expression whereas 10 μg induced a dowregulation of miR-206. Bars represent mean ± SEM. # *p *< 0.001 for comparing different capsaicin-injected doses, * *p *< 0.05, ** *p *< 0.01, and *** *p *< 0.001 for capsaicin-injected animals compared to vehicle injected mice, Mann-Whitney *U *test, n = 6 - 8,. Student's *t*-test was used for gene expression analysis where * *p *< 0.05, ** *p *< 0.01, and *** *p *< 0.001 for capsaicin-injected animals compared to vehicle injected mice, n = 6 - 8 per group.

Acute noxious stimulation may also change gene expression, and therefore we evaluated the effect of capsaicin stimulation on miRNAs regulation. Capsaicin was injected at 2 or 10 μg/20 μl in order to investigate whether the stimulus intensity correlates with a specific gene expression pattern. Thus, capsaicin administration induced a concentration-dependent nocifensive response when compared to vehicle injected mice (Figure 4A). Moreover, capsaicin induced a significant upregulation of miR-1 and miR-16, but not miR-206, in DRG (Figure 4B). However, no difference was observed between the two concentrations injected. On the other hand, no significant change was observed in the spinal dorsal horn after injecting the lower dose of capsaicin. In contrast, higher capsaicin dose reduced only miR-206 expression (Figure 4C). Collectively, these observations indicate that miRNA expression is markedly modified by different pain conditions with a high degree of spatial, temporal, and stimulus-dependent specificity.

## Discussion

Previous northern blot analysis showed that miR-1, -16 and -206 are expressed in mouse cortex, cerebellum e midbrain [10,26]. Moreover, *in silico *studies predict that those miRNAs plus miR-122a target several important pain-related genes (Table 1). Our results indicate that miR-1, -16, and -206, but not miR-122a are also expressed in DRG and the dorsal horn of the spinal cord. Moreover, the expression of these miRNAs in the nociceptive system shows spatial and temporal specificity, and a marked stimulus-dependent pattern of regulation. Thus, while miRNA expression in DRG cells promptly responded to nociceptive stimulation, the spinal cord cells were less affected. A robust amount of data have shown that various chronic pain conditions result in a dramatic alteration of gene transcription and protein synthesis in DRG, spinal cord dorsal horn and brain nuclei [21,34,35]. These changes include both up and downregulation of neuropeptides, G-protein coupled receptors, growth factors and their receptors, transcription factors as well as a large number of other messenger molecules [21-23,36]. Although the mechanisms of transcriptional regulation in DRG and spinal dorsal horn under nociceptive and pathological pain remain largely unknown, our results strongly suggest an important role for miRNAs in the activity-dependent cellular plasticity underlying chronic pain. MiRNAs regulate protein synthesis through sequence-guided interactions with the target complementary mRNA blocking the transcription and translation processes [1,37]. Therefore, changes on miRNA expression following nociceptive stimulation may represent one of the earliest events underlying the phenotypic switch induced by persistent stimulation of nociceptive primary afferents, including both high threshold C- and Aδ-fibers. Over the last few years there has been a rapid and an enormous progress in cataloging hundreds of miRNA genes, determining their expression patterns, and in few cases identifying their regulatory targets [38-40]. Notwithstanding, very few data has been raised on the miRNA expression in the nociceptive system. Bai and collaborators investigated the expression of a number of miRNAs in rat trigeminal ganglion (TG) during inflammatory muscle pain [18]. The authors investigated the time-course expression of miR-10a, -29a, -98, -99a, -124a, -134, -183 following CFA injection into the rat masseter muscle. All tested miRNAs were significantly downregulated within 4 hr after CFA administration whereas at day 12, all tested miRNAs were completely recovered to a level similar to or higher than the basal level. In our mouse model, CFA also induced a sustained down regulation of miR-1 and -16 from 12 hours to 7 days post-injection in DRG. Interestingly, Bai's study reported a fluctuating pattern of expression for some miRNAs (e.g. miR-29a, and -134), switching from downregulation to upregulation and returning to the basal levels again. We have also observed this dynamic pattern of expression in DRG following CFA injection. Thus, miR-206 showed a significant down-regulation on day 1 and 7, but not on day 3 post-injection evidencing the same unsteady pattern of expression during CFA induced inflammation. Similarly to Bai, we found no expression of miR-122, neither in DRG, nor in the dorsal horn of the spinal cord. Moreover, none of the stimuli applied were able to induce its expression. Aldrich used the spinal nerve ligation model of chronic neuropathic pain to investigate the expression of miR-183 family members (miR-96, -182, and -183) in the rat DRG [19]. The authors observed a significant reduction in expression of these miRNAs in injured DRG neurons 2 weeks after nerve injury. Using the partial sciatic nerve injury model we also observed a sustained downregulation of miR-1 and -206, but not miR-16, in DRG. Moreover, there was no change on any miRNA in the spinal dorsal horn. We extended the nerve injury assay to the neuroma model of neuropathic pain. Interestingly, all miRNA investigated showed a persistent upregulation in DRG following axotomy whereas in the dorsal horn only miR-1 was steadily downregulated. Together, our data strongly reinforces the idea that miRNA expression in the nociceptive system is stimulus-dependent. Recently, the role of Dicer, a cytoplasmic ribonuclease III that generates miRNAs, in controlling inflammatory pain was investigated. By deleting Dicer in DRG neurons expressing the voltage-gated sodium channel Na_v_1.8 the authors provided evidence that small double-stranded RNAs, such as miRNAs, are important for regulating nociceptor-associated mRNA transcripts. In addition, CFA-induced mechanical allodynia and thermal hyperalgesia were abolished in conditional mutant mice [20].

**Table 1:** Pain-related target genes predicted for miR-1, -16, -122, and -206 from in silico analysis.

Putative target	NCBI reference	microRNA	PicTar score	MicroCosm score	TargetScan score	Reference
Bdnf	NM 007540	1, 16, 122, 206	12.6, 13.9, x, 8.7	x, x, 15.9, x	-0.23, x, x, -0.23	[52-54]
Mapk3	NM 011952	1, 16, 122	x, 5.9, x	x, x, 15.5	-0.21, x, x	[55]
Calm2	NM 007589	1, 122, 206	5.6, 3.3, 6.6		-0.20, x, x	[56,57]
Ngfr	NM 033217	1, 206			-0.06, x, -0.06	[58-60]
Pla2g4a	NM 008869	1, 206		17.4, 16.9	-0.34	[61-63]
Igf1	NM 010512	1, 206	3.1, 2.3		-0.47, x	[64,65]
Trpc3	NM 019510	16		16.1		[66]
Oprd1	NM 013622	122			0.01	[67,68]

Genes, and the corresponding regulatory miRNAs predicted according to PicTar (27), MicroCosm Target version 5 (internet available at http://www.ebi.ac.uk/enright-srv/microcosm), and TargetScan (28) databases. For each predicted gene the NCBI number, predicted microRNA, PicTar score, MicroCosm score, TargetScan score, and pain-related references are provided. Larger PicTar scores, higher MicroCosm scores, and more negative TargetScan scores indicate high-likelihood of encoding a target containing conserved 3' UTR sequences; x corresponds to non-identified target in the database.

We have also addressed the question whether miRNAs expression would be influenced by acute nociceptive stimulation. Bai and collaborators have shown a significant down-regulation of a number of miRNAs as early as 30 min after CFA injection [18]. We challenge this short-time effect on miRNA expression by injecting capsaicin into the dorsal aspect of the mouse hind paw. Capsaicin immediately depolarizes primary afferent sensory neurons through the transient receptor potential vanilloid type-1, a non-selective cation channel [41]. Interestingly, 10 min after capsaicin injection miR-1 and -16 were up-regulated in DRG. A possible mechanism underlying this phenomenon might involve regulation of immediate-early genes (IEGs), such as c-*fos*, c-*jun *and c-*myc*. These genes show rapid and transient expression in the absence of *de novo *protein synthesis [42-44]. In particular, c-*fos*, which is expressed at low levels in the intact brain under basal conditions, is stereotypically induced in response to several extracellular signals, including ions, neurotransmitters, growth factors and drugs [45-47]. It is widely accepted that regulatory IEGs are involved in the stimulus-transcription coupling where c-*fos *has been considered a generic marker of neuronal depolarization [48-51]. C-Fos protein forms transcriptionally active dimmers with members of the c-*jun *family, referred to as AP-1 transcription factor. Recent data have associated miRNA activity with Fos mRNA, inhibiting Fos translation [14]. Given the importance of AP-1 as potent transcriptional activator, it is reasonable to speculate that various mechanisms would have evolved to regulate its activity, including miRNA activity. We were also interested in investigating whether miRNA expression would be dependent on stimulus intensity. Behaviorally, capsaicin administration induces a pronounced nocifensive response in a concentration-dependent manner. However, the enhanced miRNA expression in DRG did not show any association between stimulus intensity and expression pattern suggesting a ceiling effect. Conversely, in the spinal dorsal horn 10 μg, but not 2 μg, of capsaicin induced a significant downregulation of miR-206 indicating that miRNAs may also be activated in a stimulus intensity-dependent fashion.

## Conclusions

In summary, our data shows that miRNAs are differentially regulated under chronic and acute pain states. We speculate that miRNAs may be involved in the mechanisms underlying different pain conditions by fine-tuning the expression of pro and/or antinociceptive molecules. Whether these miRNAs activity is associated with the mechanisms underlying inflammatory and neuropathic pain cannot be addressed by the present study. The answer to this important question relies primarily on the elucidation of their target mRNAs. However, miRNA may integratedly modulate several genes associated with both the nociceptive and analgesic systems, influencing the dramatic neuronal changes responsible for the development and maintenance of chronic pain conditions. Most important, miRNA expression in the nociceptive system shows not only spatiotemporal specificity but is also stimulus-dependent.

## Abbreviations

A.M.: ante meridiem; Bdnf: brain derived neurotrophic factor; Calm2: calmodulin 2; CFA: complete Freund's adjuvant; DRG: dorsal root ganglion; IEG: immediate early gene; Igf1: insulin-like growth factor 1; Mapk3: mitogen-activated protein kinase 3; miRNA, miR: microRNA; mRNA: messenger RNA; Ngfr: nerve growth factor receptor Oprd1: opioid receptor, delta 1; Pla2g4a: phospholipase A2, group IVA (cytosolic, calcium-dependent); P.M.: post meridiem; RT-PCR: real-time reverse transcription polymerase chain reaction; RQ: relative quantification; TG: trigeminal ganglion; Trpc3: transient receptor potential cation channel, subfamily C, member 3; 3' UTR: three prime untranslated region

## Competing interests

The authors declare that they have no competing interests.

## Authors' contributions

**RK**, tissue extraction, RNA purifying, real time PCR assays, data analysis. **FC**, conducted behavioral tests, tissue extraction, real time PCR assays. **MIR**, data analysis, and manuscript drafting. **TAS**, RNA purification, reverse transcriptase and real time PCR assays. **SZ**, tissue extraction, RNA purifying, reverse transcriptase and real time PCR assays. **FLL**, study design, and data analysis. **GL**, study design, coordinated the project, data analysis and wrote the manuscript. All authors read and approved the final manuscript.

## References

[B1] Bartel DP MicroRNAs: genomics, biogenesis, mechanism, and function Cell 2004 116 281 297 10.1016/S0092-8674(04)00045-5 14744438

[B2] He L Hannon GJ MicroRNAs: small RNAs with a big role in gene regulation Nat Rev Genet 2004 5 522 531 10.1038/nrg1379 15211354

[B3] Kosik KS Krichevsky AM The Elegance of the MicroRNAs: A Neuronal Perspective Neuron 2005 47 779 782 10.1016/j.neuron.2005.08.019 16157272

[B4] Mattick JS Makunin IV Small regulatory RNAs in mammals Hum Mol Genet 2005 14 1 R121 132 10.1093/hmg/ddi101 15809264

[B5] Mehler MF Mattick JS Non-coding RNAs in the nervous system J Physiol 2006 575 333 341 10.1113/jphysiol.2006.113191 16809366 PMC1819441

[B6] Lee RC Feinbaum RL Ambros V The C. elegans heterochronic gene lin-4 encodes small RNAs with antisense complementarity to lin-14 Cell 1993 75 843 854 10.1016/0092-8674(93)90529-Y 8252621

[B7] Ambros V microRNAs: tiny regulators with great potential Cell 2001 107 823 826 10.1016/S0092-8674(01)00616-X 11779458

[B8] Du T Zamore PD microPrimer: the biogenesis and function of microRNA Development 2005 132 4645 4652 10.1242/dev.02070 16224044

[B9] Kim VN MicroRNA biogenesis: coordinated cropping and dicing Nat Rev Mol Cell Biol 2005 6 376 385 10.1038/nrm1644 15852042

[B10] Lagos-Quintana M Rauhut R Yalcin A Meyer J Lendeckel W Tuschl T Identification of tissue-specific microRNAs from mouse Curr Biol 2002 12 735 739 10.1016/S0960-9822(02)00809-6 12007417

[B11] Schratt GM Tuebing F Nigh EA Kane CG Sabatini ME Kiebler M Greenberg ME A brain-specific microRNA regulates dendritic spine development Nature 2006 439 283 289 10.1038/nature04367 16421561

[B12] Chen CZ Li L Lodish HF Bartel DP MicroRNAs modulate hematopoietic lineage differentiation Science 2004 303 83 86 10.1126/science.1091903 14657504

[B13] Kim HK Lee YS Sivaprasad U Malhotra A Dutta A Muscle-specific microRNA miR-206 promotes muscle differentiation J Cell Biol 2006 174 677 687 10.1083/jcb.200603008 16923828 PMC2064311

[B14] Lee HJ Palkovits M Young WS miR-7b, a microRNA up-regulated in the hypothalamus after chronic hyperosmolar stimulation, inhibits Fos translation Proc Natl Acad Sci USA 2006 103 15669 15674 10.1073/pnas.0605781103 17028171 PMC1622879

[B15] Li Y Wang F Lee JA Gao FB MicroRNA-9a ensures the precise specification of sensory organ precursors in Drosophila Genes Dev 2006 20 2793 2805 10.1101/gad.1466306 17015424 PMC1619947

[B16] Yang B Lin H Xiao J Lu Y Luo X Li B Zhang Y Xu C Bai Y Wang H Chen G Wang Z The muscle-specific microRNA miR-1 regulates cardiac arrhythmogenic potential by targeting GJA1 and KCNJ2 Nat Med 2007 13 486 491 10.1038/nm1569 17401374

[B17] Lee YS Dutta A MicroRNAs: small but potent oncogenes or tumor suppressors Curr Opin Investig Drugs 2006 7 560 564 16784027

[B18] Bai G Ambalavanar R Wei D Dessem D Downregulation of selective microRNAs in trigeminal ganglion neurons following inflammatory muscle pain Mol Pain 2007 3 15 18 10.1186/1744-8069-3-15 17559665 PMC1896151

[B19] Aldrich BT Frakes EP Kasuya J Hammond DL Kitamoto T Changes in expression of sensory organ-specific microRNAs in rat dorsal root ganglia in association with mechanical hypersensitivity induced by spinal nerve ligation Neuroscience 2009 164 711 23 10.1016/j.neuroscience.2009.08.033 19699278 PMC2762008

[B20] Zhao J Lee MC Momin A Cendan CM Shepherd ST Baker MD Asante C Bee L Bethry A Perkins JR Nassar MA Abrahamsen B Dickenson A Cobb BS Merkenschlager M Wood JN Small RNAs control sodium channel expression, nociceptor excitability, and pain thresholds J Neurosci 2010 30 10860 10871 10.1523/JNEUROSCI.1980-10.2010 20702715 PMC6634685

[B21] Hokfelt T Zhang X Wiesenfeld-Hallin Z Messenger plasticity in primary sensory neurons following axotomy and its functional implications Trends Neurosci 1994 17 22 30 10.1016/0166-2236(94)90031-0 7511846

[B22] Honore P Rogers SD Schwei MJ Salak-Johnson JL Luger NM Sabino MC Clohisy DR Mantyh PW Murine models of inflammatory, neuropathic and cancer pain each generates a unique set of neurochemical changes in the spinal cord and sensory neurons Neuroscience 2000 98 585 598 10.1016/S0306-4522(00)00110-X 10869852

[B23] Honore P Schwei J Rogers SD Salak-Johnson JL Finke MP Ramnaraine ML Clohisy DR Mantyh PW Cellular and neurochemical remodeling of the spinal cord in bone cancer pain Prog Brain Res 2000 129 389 397 full_text 11098706 10.1016/s0079-6123(00)29030-4

[B24] Kim SK Nam JW Rhee JK Lee WJ Zhang BT miTarget: microRNA target gene prediction using a support vector machine BMC Bioinformatics 2006 7 411 10.1186/1471-2105-7-411 16978421 PMC1594580

[B25] Rodriguez Parkitna J Korostynski M Kaminska-Chowaniec D Obara I Mika J Przewlocka B Przewlocki R Comparison of gene expression profiles in neuropathic and inflammatory pain J Physiol Pharmacol 2006 57 401 414 17033093

[B26] Lagos-Quintana M Rauhut R Meyer J Borkhardt A Tuschl T New microRNAs from mouse and human RNA 2003 9 175 179 10.1261/rna.2146903 12554859 PMC1370382

[B27] Krek A Grun D Poy MN Wolf R Rosenberg L Epstein EJ MacMenamin P da Piedade I Gunsalus KC Stoffel M Rajewsky N Combinatorial microRNA target predictions Nat Genet 2005 37 495 500 10.1038/ng1536 15806104

[B28] Lewis BP Shih IH Jones-Rhoades MW Bartel DP Burge CB Prediction of mammalian microRNA targets Cell 2003 115 787 798 10.1016/S0092-8674(03)01018-3 14697198

[B29] Zimmermann M Ethical guidelines for investigations of experimental pain in conscious animals Pain 1983 16 109 110 10.1016/0304-3959(83)90201-4 6877845

[B30] Seltzer Z Dubner R Shir Y A novel behavioral model of neuropathic pain disorders produced in rats by partial sciatic nerve injury Pain 1990 43 205 218 10.1016/0304-3959(90)91074-S 1982347

[B31] Dixon WJ Efficient analysis of experimental observations Annu Rev Pharmacol Toxicol 1980 20 441 462 10.1146/annurev.pa.20.040180.002301 7387124

[B32] Chaplan SR Bach FW Pogrel JW Chung JM Yaksh TL Quantitative assessment of tactile allodynia in the rat paw J Neurosci Methods 1994 53 55 63 10.1016/0165-0270(94)90144-9 7990513

[B33] Livak KJ Schmittgen TD Analysis of relative gene expression data using real-time quantitative PCR and the 2(-Delta Delta C(T)) Method Methods 2001 25 402 408 10.1006/meth.2001.1262 11846609

[B34] Alvares D Fitzgerald M Building blocks of pain: the regulation of key molecules in spinal sensory neurones during development and following peripheral axotomy Pain 1999 6 Suppl S71 85 10.1016/S0304-3959(99)00140-2 10491975

[B35] Woolf CJ Phenotypic modification of primary sensory neurons: the role of nerve growth factor in the production of persistent pain Philos Trans R Soc Lond B Biol Sci 1996 351 441 448 10.1098/rstb.1996.0040 8730783

[B36] Mannion RJ Costigan M Decosterd I Amaya F Ma QP Holstege JC Ji RR Acheson A Lindsay RM Wilkinson GA Woolf CJ Neurotrophins: peripherally and centrally acting modulators of tactile stimulus-induced inflammatory pain hypersensitivity Proc Natl Acad Sci USA 1999 96 9385 9390 10.1073/pnas.96.16.9385 10430952 PMC17792

[B37] Liu J Valencia-Sanchez MA Hannon GJ Parker R MicroRNA-dependent localization of targeted mRNAs to mammalian P-bodies Nat Cell Biol 2005 7 719 723 10.1038/ncb1274 15937477 PMC1855297

[B38] Fabian MR Sonenberg N Filipowicz W Regulation of mRNA translation and stability by microRNAs Annu Rev Biochem 2010 79 351 379 10.1146/annurev-biochem-060308-103103 20533884

[B39] Herranz H Cohen SM MicroRNAs and gene regulatory networks: managing the impact of noise in biological systems Genes Dev 2010 24 1339 1344 10.1101/gad.1937010 20595229 PMC2895193

[B40] Newman MA Hammond SM Emerging paradigms of regulated microRNA processing Genes Dev 2010 24 1086 1092 10.1101/gad.1919710 20516194 PMC2878647

[B41] Caterina MJ Schumacher MA Tominaga M Rosen TA Levine JD Julius D The capsaicin receptor: a heat-activated ion channel in the pain pathway Nature 1997 389 816 824 10.1038/39807 9349813

[B42] Barth AL Visualizing circuits and systems using transgenic reporters of neural activity Curr Opin Neurobiol 2007 17 567 571 10.1016/j.conb.2007.10.003 18036810 PMC2696220

[B43] Kovacs KJ Measurement of immediate-early gene activation-c-fos and beyond J Neuroendocrinol 2008 20 665 672 10.1111/j.1365-2826.2008.01734.x 18601687

[B44] Sng JC Taniura H Yoneda Y A tale of early response genes Biol Pharm Bull 2004 27 606 612 10.1248/bpb.27.606 15133230

[B45] Coggeshall RE Fos, nociception and the dorsal horn Prog Neurobiol 2005 77 299 352 16356622 10.1016/j.pneurobio.2005.11.002

[B46] Lima D Almeida A The medullary dorsal reticular nucleus as a pronociceptive centre of the pain control system Prog Neurobiol 2002 66 81 108 10.1016/S0301-0082(01)00025-9 11900883

[B47] Zhao ZQ Neural mechanism underlying acupuncture analgesia Prog Neurobiol 2008 85 355 375 10.1016/j.pneurobio.2008.05.004 18582529

[B48] Herdegen T Zimmermann M Immediate early genes (IEGs) encoding for inducible transcription factors (ITFs) and neuropeptides in the nervous system: functional network for long-term plasticity and pain Prog Brain Res 1995 104 299 321 full_text 8552775 10.1016/s0079-6123(08)61797-5

[B49] Loebrich S Nedivi E The function of activity-regulated genes in the nervous system Physiol Rev 2009 89 1079 103 10.1152/physrev.00013.2009 19789377 PMC2828052

[B50] Harris JA Using c-fos as a neural marker of pain Brain Res Bull 1998 45 1 8 10.1016/S0361-9230(97)00277-3 9434195

[B51] Zimmermann M Immediate-early genes in the nervous system-are they involved in mechanisms of chronic pain? Patol Fiziol Eksp Ter 1992 4 47 51 1303503

[B52] Kerr BJ Bradbury EJ Bennett DL Trivedi PM Dassan P French J Shelton DB McMahon SB Thompson SW Brain-derived neurotrophic factor modulates nociceptive sensory inputs and NMDA-evoked responses in the rat spinal cord J Neurosci 1999 19 5138 5148 10366647 10.1523/JNEUROSCI.19-12-05138.1999 PMC6782650

[B53] Merighi A Salio C Ghirri A Lossi L Ferrini F Betelli C Bardoni R BDNF as a pain modulator Prog Neurobiol 2008 85 297 317 10.1016/j.pneurobio.2008.04.004 18514997

[B54] Thompson SW Bennett DL Kerr BJ Bradbury EJ McMahon SB Brain-derived neurotrophic factor is an endogenous modulator of nociceptive responses in the spinal cord Proc Natl Acad Sci USA 1999 96 7714 7718 10.1073/pnas.96.14.7714 10393886 PMC33607

[B55] Galan A Lopez-Garcia JA Cervero F Laird JM Activation of spinal extracellular signaling-regulated kinase-1 and -2 by intraplantar carrageenan in rodents Neurosci Lett 2002 322 37 40 10.1016/S0304-3940(02)00078-2 11958838

[B56] Wei F Qiu CS Kim SJ Muglia L Maas JW Pineda VV Xu HM Chen ZF Storm DR Muglia LJ Zhuo M Genetic elimination of behavioral sensitization in mice lacking calmodulin-stimulated adenylyl cyclases Neuron 2002 36 713 726 10.1016/S0896-6273(02)01019-X 12441059

[B57] Zeitz KP Giese KP Silva AJ Basbaum AI The contribution of autophosphorylated alpha-calcium-calmodulin kinase II to injury-induced persistent pain Neuroscience 2004 128 889 898 10.1016/j.neuroscience.2004.07.029 15464294

[B58] Davar G Shalish C Blumenfeld A Breakfield XO Exclusion of p75NGFR and other candidate genes in a family with hereditary sensory neuropathy type II Pain 1996 67 135 139 10.1016/0304-3959(96)03113-2 8895241

[B59] Fukui Y Ohtori S Yamashita M Yamauchi K Inoue G Suzuki M Orita S Eguchi Y Ochiai N Kishida S Takaso M Wakai K Hayashi Y Aoki Y Takahashi K Low affinity NGF receptor (p75 neurotrophin receptor) inhibitory antibody reduces pain behavior and CGRP expression in DRG in the mouse sciatic nerve crush model J Orthop Res 2009 28 279 283 10.1002/jor.20986 19824062

[B60] Watanabe T Ito T Inoue G Ohtori S Kitajo K Doya H Takahashi K Yamashita T The p75 receptor is associated with inflammatory thermal hypersensitivity J Neurosci Res 2008 86 3566 3574 10.1002/jnr.21808 18709654

[B61] Hasegawa S Kohro Y Shiratori M Ishii S Shimizu T Tsuda M Inoue K Role of PAF receptor in proinflammatory cytokine expression in the dorsal root ganglion and tactile allodynia in a rodent model of neuropathic pain PLoS One 2010 5 e10467 10.1371/journal.pone.0010467 20454616 PMC2862737

[B62] Inoue M Ma L Aoki J Ueda H Simultaneous stimulation of spinal NK1 and NMDA receptors produces LPC which undergoes ATX-mediated conversion to LPA, an initiator of neuropathic pain J Neurochem 2008 107 1556 1565 10.1111/j.1471-4159.2008.05725.x 19014389

[B63] Yeo JF Ong WY Ling SF Farooqui AA Intracerebroventricular injection of phospholipases A2 inhibitors modulates allodynia after facial carrageenan injection in mice Pain 2004 112 148 155 10.1016/j.pain.2004.08.009 15494195

[B64] Contreras PC Vaught JL Gruner JA Brosnan C Steffler C Arezzo JC Lewis ME Kessler JA Apfel SC Insulin-like growth factor-I prevents development of a vincristine neuropathy in mice Brain Res 1997 774 20 26 10.1016/S0006-8993(97)81682-4 9452187

[B65] de Pablo F Banner LR Patterson PH IGF-I expression is decreased in LIF-deficient mice after peripheral nerve injury Neuroreport 2000 11 1365 1368 10.1097/00001756-200004270-00043 10817623

[B66] Staaf S Oerther S Lucas G Mattsson JP Ernfors P Differential regulation of TRP channels in a rat model of neuropathic pain Pain 2009 144 187 199 10.1016/j.pain.2009.04.013 19446956

[B67] Gaveriaux-Ruff C Karchewski LA Hever X Matifas A Kieffer BL Inflammatory pain is enhanced in delta opioid receptor-knockout mice Eur J Neurosci 2008 27 2558 2567 10.1111/j.1460-9568.2008.06223.x 18513322 PMC4445739

[B68] Nadal X Banos JE Kieffer BL Maldonado R Neuropathic pain is enhanced in delta-opioid receptor knockout mice Eur J Neurosci 2006 23 830 834 10.1111/j.1460-9568.2006.04569.x 16487163

